# Disease-associated HCN4 V759I variant is not sufficient to impair cardiac pacemaking

**DOI:** 10.1007/s00424-020-02481-3

**Published:** 2020-10-23

**Authors:** Nadine Erlenhardt, Olaf Kletke, Franziska Wohlfarth, Marlene A. Komadowski, Lukas Clasen, Hisaki Makimoto, Susanne Rinné, Malte Kelm, Christiane Jungen, Niels Decher, Christian Meyer, Nikolaj Klöcker

**Affiliations:** 1 Institute of Neurophysiology, Medical Faculty, University of Düsseldorf, Universitätsstr 1, 40225 Düsseldorf, Germany; 2 Institute of Physiology and Pathophysiology and Marburg Center for Mind, Brain, and Behavior, Philipps-University Marburg, Deutschhausstrasse 1-2, 35037 Marburg, Germany; 3 Department of Cardiology Pulmonology and Vascular Medicine Medical Faculty, University Hospital Düsseldorf, Moorenstr 5, 40225 Düsseldorf, Germany; 4 Department of Cardiology, University Heart Center, University Hospital Hamburg-Eppendorf, Martinistr 52, 20246 Hamburg, Germany; 5 DZHK (German Center for Cardiovascular Research), Berlin, Germany; 6 Present Address: Department of Cardiology Willem Einthoven Center for Cardiac Arrhythmia Research and Management, Leiden University Medical Center, Albinusdreef 2, Leiden, Netherlands; 7 Division of Cardiology, cardiac Neuro- and Electrophysiology Research Consortium (cNEP), EVK Düsseldorf, Kirchfeldstrasse 40, 40217 Düsseldorf, Germany

**Keywords:** Sick sinus syndrome, HCN channel, PEX5R, Trip8b, Funny current, Genetic variant

## Abstract

The hyperpolarization-activated cation current *I*_*f*_ is a key determinant for cardiac pacemaker activity. It is conducted by subunits of the hyperpolarization-activated cyclic nucleotide–gated (HCN) channel family, of which HCN4 is predominant in mammalian heart. Both loss-of-function and gain-of-function mutations of the HCN4 gene are associated with sinus node dysfunction in humans; however, their functional impact is not fully understood yet. Here, we sought to characterize a HCN4 V759I variant detected in a patient with a family history of sick sinus syndrome. The genomic analysis yielded a mono-allelic HCN4 V759I variant in a 49-year-old woman presenting with a family history of sick sinus syndrome. This HCN4 variant was previously classified as putatively pathogenic because genetically linked to sudden infant death syndrome and malignant epilepsy. However, detailed electrophysiological and cell biological characterization of HCN4 V759I in *Xenopus laevis* oocytes and embryonic rat cardiomyocytes, respectively, did not reveal any obvious abnormality. Voltage dependence and kinetics of mutant channel activation, modulation of cAMP-gating by the neuronal HCN channel auxiliary subunit PEX5R, and cell surface expression were indistinguishable from wild-type HCN4. In good agreement, the clinically likewise affected mother of the patient does not exhibit the reported HCN4 variance. HCN4 V759I resembles an innocuous genetic HCN channel variant, which is not sufficient to disturb cardiac pacemaking. Once more, our work emphasizes the importance of careful functional interpretation of genetic findings not only in the context of hereditary cardiac arrhythmias.

## Introduction

The sick sinus syndrome (SSS) is a heterogeneous group of disorders, in which the heart fails to perform adequate pacemaking function. It is of great socioeconomic relevance as it represents the most common indication for pacemaker implantation accounting for up to 50% of implant diagnoses [16]. Clinically, the SSS subsumes a variety of manifestations ranging from symptomatic sinus bradycardia, chronotropic incompetence, sinuatrial block, and sinus arrest, to atrial ectopies and paroxysmal supraventricular tachyarrhythmias, all reflecting a dysfunction of the sinuatrial node (SAN) in either the formation of the electrical impulse or in its conduction to the atrial myocardium [19]. The pathogenesis of SSS can be just as diverse as the clinical  presentation: besides acquired and congenital structural heart disease, metabolic disorders, and drug therapy, also hereditary forms of SSS have been reported. In the latter, channelopathies have been identified as a putative pathomechanism [22, 31], with loss-of-function and gain-of-function mutations in the cardiac pacemaker channel HCN4 making up for most of the reported familial forms of SSS.

Hyperpolarization-activated cyclic nucleotide–gated (HCN) channels form the ion pore conducting the cardiac pacemaker current [9]. As its activation by hyperpolarization is unique among all other known voltage-dependent currents, it was termed “funny” (*I*_*f*_). A further hallmark of *I*_*f*_ is its direct facilitation by cyclic nucleotides [3]. Regulation of the intracellular concentration of cAMP may therefore control the availability of *I*_*f*_ and is thought to contribute to the autonomic regulation of heart rate. Recent experimental evidence indicates that, besides its well-known role in generating pacemaking activity, *I*_*f*_ is required for SAN cell synchronization, impulse propagation, and resulting in regular beat-to-beat variation [32]. Four isoforms of the mammalian HCN channel family of proteins have been cloned (HCN1–4) [3]. Heterologous expression of HCN subunits reconstitutes the major hallmarks of *I*_*f*_ including the activation by hyperpolarization, its facilitation by cyclic nucleotides, and its ion selectivity [2]. The four HCN isoforms are differentially expressed in the heart tissue. In mammals, including humans, HCN4 is the predominant isoform in the SAN [4].

Here, we describe the case of a 49-year-old woman with a family history of SSS. The genomic analysis yielded a mono-allelic HCN4 V759I variant. The HCN4 mutation V759I had been previously classified as putatively pathogenic as it was found in cases of sudden unexpected death in epilepsy (SUDEP) and sudden infant death syndrome (SIDS) [12, 29, 31]. However, both clinical and mechanistic data on the putative impact of this HCN4 variant have been lacking so far.

## Methods

### Clinical records

The studies in human subjects were in accordance with the ethical standards in the revised version of the Declaration of Helsinki and approved by the local ethics committee (# 4869). Informed written consent was given prior to the inclusion of subjects in the study.

### Molecular biology

Genomic DNA was extracted from whole blood using the QIAmp DNA Blood Mini Kit (Qiagen) following the supplier’s instructions. All HCN4 exons were amplified by PCR using intronic HCN4-specific primers [25], 125 ng genomic DNA, 5–8% DMSO, and Phusion DNA Polymerase (Thermo Fisher Scientific). PCR conditions were 30 s at 98 °C (1 cycle), 10 s at 98 °C, 30 s at 60–70 °C, 15 s at 72 °C (30 cycles), and 2 min at 72 °C (1 cycle). Amplificates were separated by agarose gel electrophoresis, purified with the MinElute Gel Extraction Kit (Qiagen), and analyzed by Sanger sequencing. The electropherograms were compared to wild-type human HCN4 using VectorNTI software (Life Technologies).

In vitro transcription and injection of cRNAs into *Xenopus laevis* oocytes was performed as described before [37]. Site-directed mutagenesis was done as previously described [15]. GenBank accession numbers of cDNAs were NM_005477 (human HCN4) and NM_173152 (rat PEX5R/Trip8b). All cDNAs were verified by sequencing.

### Electrophysiological recordings

*X. laevis* oocytes were prepared using standard methods as described previously [26, 37]. Procedures conform to the Directive 2010/63/EU of the European Parliament and were approved by the local Animal Welfare Committee (AZ 84-02.05.20.12.136). Briefly, ovarian lobes were surgically removed in aseptic conditions from adult female *X. laevis* frogs anesthetized in ice water with 0.1% tricaine (MS-222). After surgery, frogs were allowed to recover to consciousness followed by at least 3 months of recovery without surgery. Oocyte collection was alternated between left and right ovaries with a maximum of four surgeries in one individual animal. After final surgery, the anesthetized frog was euthanized by decapitation. Twenty-four hours after surgery, stages V–IV oocytes were selected, injected with 5–15 ng cRNA per oocyte, and incubated in Barth^+^ (in mM: 88 NaCl, 1 KCl, 0.4 CaCl_2_, 0.33 Ca(NO_3_)_2_, 2.4 NaHCO_3_, 0.8 MgSO_4_, 5 HEPES, pH 7.2), supplemented with 100 U/ml penicillin, 100 U/ml streptomycin from an antibiotic antimycotic solution (× 100) (Sigma-Aldrich) at 18 °C. After 2–7 days, transmembrane currents could be recorded using the two-electrode voltage clamp (TEVC) technique. Electrodes with a tip resistance of 0.5–1 MΩ were prepared using a DMZ-Universal micropipette puller (Zeitz Instruments) and filled with 3 M KCl. Oocytes were placed in a custom-made chamber and superfused with HCN Ringer solution (in mM: 17.5 NaCl, 115 KCl, 1.8 CaCl_2_, 10 Hepes, pH 7.3). Current signals were recorded using a TEVC amplifier (TURBO TEC-03X, npi) coupled to a LIH8+8 computer interface (HEKA), and stored using Patchmaster v2 × 65 (HEKA). All experiments were performed at room temperature (RT). Steady-state activation curves were determined with a tail current protocol. Briefly, preconditioning voltage steps (from a holding potential of − 20 mV) were applied to potentials between − 20 mV and − 150 mV (10 mV increments) for a duration of 5 s before the membrane potential was stepped to − 150 mV for 2 s to elicit the tail current. Currents recorded at the tail potential were normalized to maximum, plotted versus the preconditioning potential, and fitted with a Boltzmann function. Curve fitting and further data analysis were done with Patchmaster and Fitmaster software (HEKA). Data are given as mean ± SEM.

Inside-out macropatch clamp recordings from *X. laevis* oocytes were performed as previously described [27]. Briefly, patch pipettes were pulled from thick-walled borosilicate glass capillaries BF200-100-10 (Science Products GmbH, Hofheim, Germany) and had tip resistances between 0.3 and 1.0 MΩ. Inside-out macropatch clamp experiments were conducted with a pipette solution containing (in mM): KCl 120, HEPES 10, and CaCl_2_ 1.0; and adjusted to pH 7.2 with KOH. Macropatches were perfused with a bath solution composed of (in mM): KCl 120, HEPES 10, EGTA 10; and adjusted to pH 7.2 with KOH. For voltage-dependence of activation experiments, the bath solution was supplemented with 100 μM 3′,5′-cAMP (Sigma-Aldrich) to analyze the shift caused by cAMP. Bath solutions were applied via a 4-channel valve controlled gravity perfusion system (ALA Scientific Instruments, New York, USA) to the cytoplasmic side of excised membrane patches. All patch-clamp recordings were made using an Axopatch 200B amplifier (Molecular Devices, Sunnyvale CA, USA) and Clampex 10.6 software (Molecular Devices, Sunnyvale CA, USA). Data were digitized with a Digidata 1550B digitizer (Molecular Devices, Sunnyvale CA, USA) and filtered at 5 kHz. Data were analyzed using Microsoft Excel and OriginPro (OriginLab Corp., Guangzhou, China). Statistical significance was calculated using the unpaired Student’s *t* test. All experiments were performed at room temperature (21–24 °C). Tail currents were obtained in response to 1 s voltage steps to potentials between − 60 mV and − 160 mV (increment of − 10 mV). The holding potential was 0 mV and tail currents were recorded at + 50 mV. Tail currents were normalized for each measurement and the *V*_1/2_ of conductance–voltage relationships (GVs) were determined by fitting the data to a Boltzmann equation.

Based on the weak selectivity of HCN channels for K^+^ over Na^+^ (P_K+_/P_Na+_: 3–5) and the reported dependence of channel conductance on extracellular K^+^ [8, 14, 34], the electrophysiological experiments were performed in high K^+^/low or no Na^+^ to enhance signal-to-noise.

### Cell culture, viral gene transfer, immunocytochemistry

Cardiomyocytes were prepared from hearts of E18 embryonic Wistar rats. Procedures conform to the Directive 2010/63/EU of the European Parliament and were approved by the local Animal Welfare Committee (O7/11, AZ 81-02.04.2018.A389). Briefly, pregnant mother animals were euthanized by decapitation after isoflurane pre-anesthesia. E18 embryos were quickly dissected and also euthanized by decapitation. Embryonic hearts were cut into small pieces and digested with 0.05% Collagenase type 2 (Worthington) and 0.15% Trypsin. Cell suspension was filtered through a 70-μm cell strainer and cardiomyocytes were pelleted by centrifugation at 1200 rpm for 8 min. Cells were initially seeded onto gelatin-coated coverslips at a density of 60,000 cells/24-well in plating medium (DMEM + Glutamax supplemented with 20% fetal bovine serum (FBS, Biochrom), 1% non-essential amino acids, 1% penicillin/streptomycin and 0.0004% β-Mercaptoethanol (all Life Technologies)). After 24 h, cells were treated with mitomycin (10 μg/ml) for 1 h in maintenance medium (plating medium with 1% FBS) and afterwards cultured in maintenance medium. The medium was replaced once daily. Cardiomyocytes were identified by the striated aspect of their actin cytoskeleton stained with AF647-conjugated phalloidin (Life Technologies).

At DIV2–3, cardiomyocytes were infected with recombinant Semliki Forest Virus (SFV) for expression of wild-type or V759I variant HCN4 tagged with an extracellular hemagglutinin (HA) epitope [15]. To this end, HCN4 constructs were inserted into pSFV. For virus production, pSFV cDNAs and a helper plasmid were linearized, followed by in vitro transcription using the mMESSAGE mMACHINE SP6 Transcription Kit (Thermo Fisher Scientific). Baby hamster kidney cells (BHK-21) cultured in MEM + Glutamax supplemented with 5% FBS and 1% penicillin/streptomycin and grown to 80% confluence were electroporated with purified cRNAs of the transgene and the helper plasmid. After incubating electroporated BHK-21 cells at 37 °C and 5% CO_2_ for 48 h, the culture medium containing the viral particles was harvested, purified, and stored at − 80 °C. For infection of cardiomyocytes, the virus was activated by 45 min incubation with chymotrypsin (0.5 mg/ml) followed by 10 min incubation with aprotinin (0.67 mg/ml).

Sixteen to 20 h after viral transduction, the surface population of wild-type and variant HCN4 channels were detected by incubation of living cardiomyocytes with a mouse monoclonal anti-HA antibody (1:100, Santa Cruz) in DMEM + Glutamax for 30 min at 37 °C followed by a brief washing step in phosphate-buffered saline (PBS) and subsequent incubation with secondary goat anti-mouse antiserum conjugated to cy3 (1:500, Life Technologies) for 30 min at 37 °C. For detection of intracellular HCN4 channels, cells were fixed with 4% paraformaldehyde, permeabilized, and blocked with 10% normal goat serum in PBS containing 0.01% Triton X-100 and incubated with the mouse monoclonal anti-HA antibody (1:100) for 1 h at RT followed by incubation with goat anti-mouse antiserum conjugated to AF488 (1:500, 1 h, Life Technologies). Coverslips were mounted onto microscope slides using ProLong Gold Antifade Mountant (Life Technologies). Image acquisition and quantification of fluorescence intensities were performed as described in Milstein et al. [18] using a LSM510 Meta microscope (Zeiss) and ImageJ software (NIH).

## Results

### Case report

**Fig. 1:**
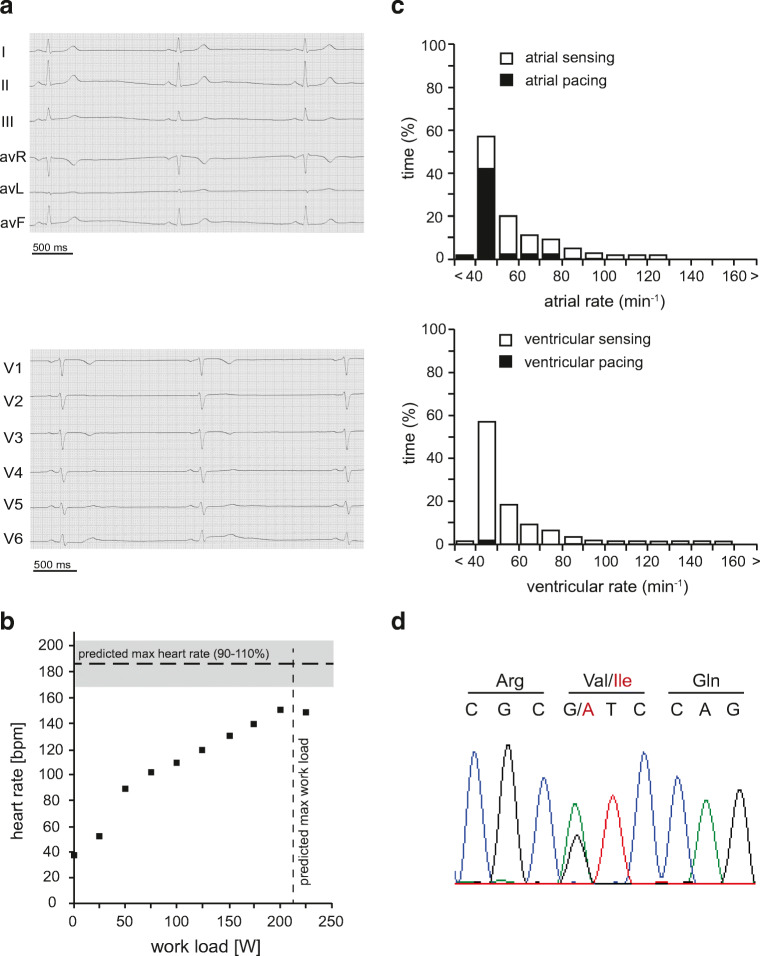
Detection of the disease-associated HCN4 V759I variant in a 49-year-old patient with sick sinus syndrome. **a** Baseline 12-lead electrocardiogram of the patient, who suffered from dizziness and nausea, showing sinus bradycardia (37 bpm, cycle length 1600 ms) without any evidence for additional disturbances of cardiac depolarization or repolarization (PR-interval, 140 ms; QRS, 100 ms; QT-interval, 440 ms (QT_*c*_, 348 ms)). **b** Patient’s heart rates during bicycle exercise before pacemaker implantation. Predicted maximum heart rate (reference interval, 90–110%) and predicted maximum workload corrected for age are indicated by horizontal and vertical dotted lines, respectively [13]. **c** Atrial and ventricular frequency histograms from pacemaker interrogation two months after implantation showing a proportion of 42.3% atrial and < 0.1% ventricular pacing (lower intervention frequency: 45 bpm). **d** Direct sequencing of all exons of the HCN4 gene revealed heterozygous variance in exon 8, which results in the single amino acid exchange V759I

A 49-year-old woman presented with repeated episodes of dizziness and nausea at our outpatient clinic. On admission, marked sinus bradycardia of 37 bpm was documented. Electrocardiographic (ECG) examination did not reveal any disturbances of cardiac depolarization or repolarization (Fig. 1 a); the PR, QRS, and QT intervals were in the normal reference range (PR 140 ms; QRS 100 ms; QT 440 ms (QT_*c*_ 348 ms)). During exercise electrocardiography, the patient showed sinus rhythm and reached a maximum heart rate of 146 bpm at a maximum workload of 225 W (Fig. 1b). This was below the predicted heart rate for respective age and gender [13]. A 24 h ECG showed heart rates of 27–117 bpm, two sinus pauses of up to 3 s, and intermittent bradycardia–related bigeminus and trigeminus, but no ventricular tachycardias. The calculated intrinsic heart rate was 89 bpm. In the absence of detectable heart disease, the patient was diagnosed with a symptomatic sick sinus syndrome (SSS), and a dual-chamber pacemaker was implanted. During follow-up, the patient’s symptoms had improved and pacemaker interrogation revealed proportions of predominant atrial pacing (42.3%) with intrinsic atrioventricular and sequential ventricular activity at a lower intervention frequency of 45 bpm (Fig. 1c).

As the patient reported a family history of SSS, we sequenced all eight exons of the HCN4 gene and identified a mono-allelic non-synonymous variant of c.2275G>A in exon 8 resulting in the single amino acid exchange of V759I in the translated protein. HCN4 V759I has been described as a rare variant in a European population at a frequency of 0.6% and in an African population at 0.1% (National Heart, Lung, and Blood Institute Exome Variant Server, EVS, http://evs.gs.washington.edu/EVS/). The mutation V759I in HCN4 has been linked to cases of sudden unexpected death in epilepsy (SUDEP) and sudden infant death syndrome (SIDS) [12, 29]. It has therefore been classified as likely pathogenic [31].

The mother of the patient reported palpitations since the age of 64 without any evidence for structural heart disease. Pacemaker implantation was performed in 2014 after detection of symptomatic sinus pauses of 4 s during 24-h Holter monitoring. Pacemaker interrogation revealed predominant bradycardia at a lower intervention frequency of 60 bpm. Proportions of dual-chamber pacing were equally high due to sinus bradycardia and prolonged atrioventricular conduction time of 300 ms (atrial pacing 91%, ventricular pacing 88.1%). No abnormalities, however, were detected in the exon sequence of her HCN4 gene.

### Gating characteristics of the disease-associated variant HCN4 V759I

**Fig. 2:**
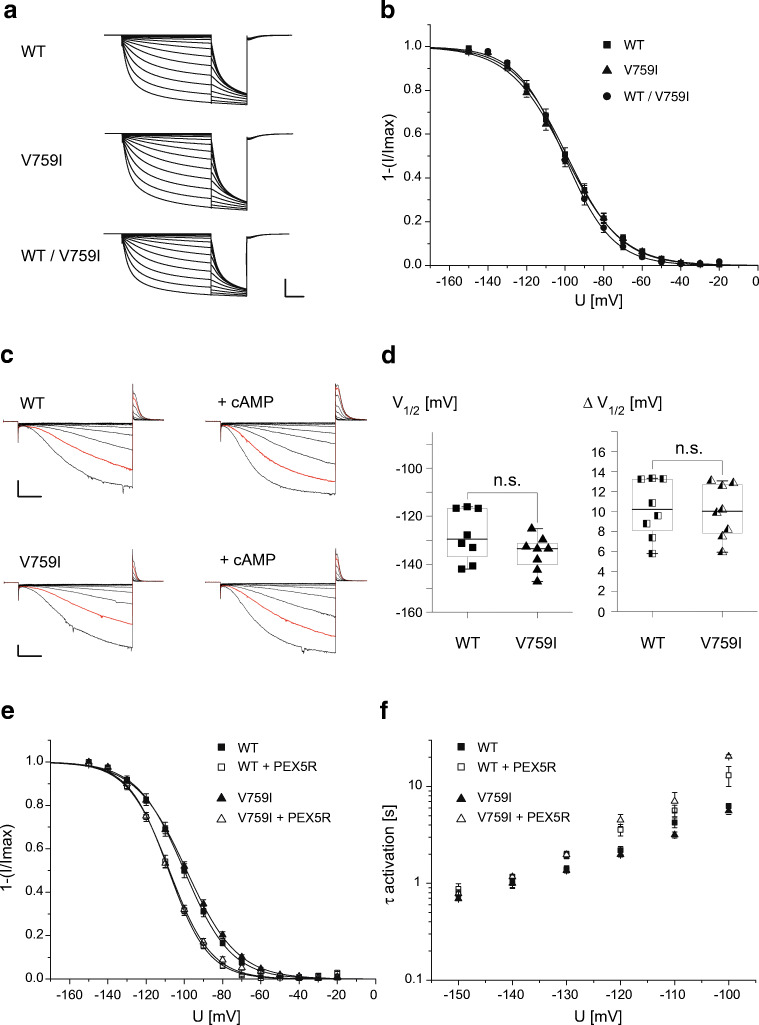
The biophysical properties of the disease-associated HCN4 V759I variant are indistinguishable from wild-type HCN4. **a** Representative currents recorded in *Xenopus laevis* oocytes expressing wild-type (WT), variant (V759I), and co-expressing both wild-type and variant HCN4 channels (WT/V759I) in response to voltage steps to potentials between − 20 and − 150 mV in 10 mV increments (holding potential − 20 mV, tail potential − 150 mV). Scale bar: 5 μA and 2 s. **b** Steady-state activation curves of wild-type and variant HCN4 channels as indicated. Data points are mean ± SEM of *n* = 12–17 oocytes of *N* = 2 experiments (individual batches of oocytes). Lines are fits of a Boltzmann function to the data with values for *V*_1/2_ of − 100.4 ± 2.0 mV (slope factor 14.0 ± 0.3 mV) for WT (*n* = 12), − 101.5 ± 1.9 mV (slope factor 14.6 ± 0.4 mV) for V759I (*n* = 12), and − 101.2 ± 1.5 mV (slope factor 12.5 ± 0.2 mV) for co-expressed WT/V759I channels (*n* = 17). **c** Representative currents recorded from inside-out patches from *X. laevis* oocytes expressing wild-type (WT) and variant (V759I) HCN4 channels in response to voltage steps to potentials between − 60 and − 160 mV in − 10 mV increments (holding potential 0 mV, tail potential + 50 mV). Traces in red are current responses to a step potential of − 150 mV. Scale bars: 75 pA, 200 ms. **d** Quantification of cAMP-mediated modulation of voltage-dependent activation of wild-type (WT) and variant (V759I) HCN4 channels in inside-out patches (Materials and Methods). Left, half-maximal activation voltages (*V*_1/2_) prior cAMP application for wild-type (WT) and variant (V759I) HCN4 channels; right, shifts of *V*_1/2_ (Δ*V*_1/2_) by application of cAMP (100 μM) in HCN4 channels as indicated. Box plots display median and 25/75 percentiles, whiskers indicate outliers. n.s., not significant (*n* = 8). **e** Steady-state activation curves of wild-type (WT) and variant (V759I) HCN4 channels with (open symbols) and without (filled symbols) co-expression of the brain HCN channel auxiliary subunit PEX5R. Data points are mean ± SEM of *n* = 6–10 oocytes of *N* = 2 TEVC experiments (individual batches of oocytes). Lines are fits of a Boltzmann function to the data with values for *V*_1/2_ of − 100.9 ± 1.6 mV (slope factor 12.3 ± 0.1 mV) for WT (*n* = 8), − 108.9 ± 1.2 mV (slope factor 10.5 ± 0.3 mV) for WT + PEX5R (*n* = 6), − 99.6 ± 1.2 mV (slope factor 13.3 ± 0.2) for V759I channels (*n* = 9), and − 109.1 ± 0.9 mV (slope 11.0 ± 0.4) for V759I + PEX5R co-expressed channels (*n* = 10). **f** Activation kinetics of wild-type (WT) and variant (V759I) HCN4 channels with (open symbols) and without (filled symbols) co-expression of the brain HCN channel auxiliary subunit PEX5R. Time constants (*τ* activation) were calculated from mono-exponential fits of current activation at indicated voltage steps (holding potential − 20 mV; *n* = 4–21 oocytes)

Given the reported association of HCN4 V759I with SUDEP and SIDS [12, 29], we sought to investigate a putative impact of the mutation on HCN4 function. Wild-type HCN4 and mutant HCN4 V759I channels were expressed in *X. laevis* oocytes [26] and first assessed by two-electrode voltage clamp (TEVC) recordings. Voltage steps to hyperpolarizing potentials gave rise to slowly activating inward currents of similar amplitude independent of the channel variant expressed (Fig. 2a; maximal current amplitudes upon injection of 10 ng cRNAs were 11.19 ± 1.42 μA (*n* = 15) and 10.44 ± 1.99 μA (*n* = 20) for HCN4 and HCN4 V759I, respectively). Voltage-dependence of channel activation of HCN4 V759I was indistinguishable from wild-type HCN4 with values for *V*_1/2_ of − 100.4 ± 2.0 mV (*n* = 12; *N* = 2) for wild-type and − 101.5 ± 1.9 mV (*n* = 12; *N* = 2) for mutant channels (Fig. 2b). Also, co-expression of HCN4 and HCN4 V759I mimicking the patient’s heterozygous genotype did not change voltage-dependent activation of wild-type channels (value for *V*_1/2_ of − 101.2 ± 1.5 mV (*n* = 17; *N* = 2)). In line with the preserved voltage-dependence of channel activation, also the kinetics of channel activation did not differ between wild-type and HCN4 V759I (Fig. 2f). In inside-out patches from *X. laevis* oocytes, modulation of voltage-dependent activation of wild-type and variant channels by the cyclic nucleotide cAMP was probed (Fig. 2c). As quantified in Fig. 2d, cAMP shifted voltage-dependent activation of both wild-type HCN4 and variant HCN4 V759I channels to the same extent, that is by 10.3 ± 1 mV (*n* = 8) and 10.0 ± 0.9 mV (*n* = 8), respectively, starting from insignificantly different activation voltages prior cAMP application (values for *V*_1/2_ of − 128.0 ± 3.7 mV and − 135.3 ± 2.5 mV for wild-type and variant HCN4, respectively, *n* = 8). As PEX5R was identified as an auxiliary subunit of HCN channel complexes in the brain [37], and the genetic variant V759I was linked to malignant epilepsy [29]; we also investigated the effects of PEX5R on mutant HCN4 channels. Figure 2e shows that co-expression of PEX5R shifted voltage-dependence of both wild-type and mutant channel activation to more hyperpolarized values of similar extent (values for *V*_1/2_ of − 100.9 ± 1.6 mV for wild-type (*n* = 8), − 108.9 ± 1.2 mV for wild-type + PEX5R (*n* = 6), − 99.6 ± 1.2 mV for V759I channels (*n* = 9), and − 109.1 ± 0.9 mV for V759I + PEX5R channels (*n* = 10)). In good agreement with the view of PEX5R as an antagonist of channel modulation by cyclic nucleotides [37], co-expression of PEX5R also slowed channel activation (Fig. 2f). The increase in time constants of channel activation was comparable in wild-type and mutant HCN4.

Thus, neither voltage-dependence, nor kinetics, nor modulation of channel activation by cyclic nucleotides are affected by the amino acid exchange V759I.

### Cell surface expression of the disease-associated variant HCN4 V759I

**Fig. 3:**
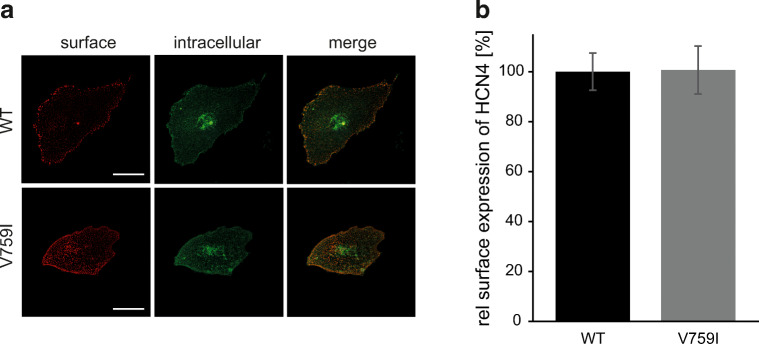
Surface trafficking of disease-associated HCN4 V759I variant is not disturbed. **a** Representative micrographs of embryonic rat cardiomyocytes (E18), 16 h after viral transduction with wild-type (WT) or variant (V759I) HCN4 with an extracellular hemagglutinin (HA) tag. Surface and intracellular populations of channels were detected by monoclonal anti-HA immunocytochemistry without and after plasma membrane permeabilization, respectively. Scale bar: 20 μM. **b** Quantification of wild-type (WT) and variant (V759I) HCN4 channel surface expression [18]. Data are given as mean ± SEM calculated as surface/(surface + intracellular) fluorescence intensities normalized to wild-type control (relative (rel) surface expression, *n* = 22 cells of *N* = 3 experiments)

Besides their biophysical gating properties, also the number of ion channels on the cell surface determines the extent of transmembrane current that can be conducted and hence their functional role. Numerous mutations have been identified in the mammalian ion channel proteome, which impair surface trafficking of channels while leaving their gating characteristics unaffected [5]. Therefore, we studied cell surface expression of wild-type and mutant HCN4 V759I protein by extracellular epitope tagging. Embryonic rat cardiomyocytes (E18) were virally transduced with wild-type and variant HCN4, both carrying an extracellular hemagglutinin (HA) epitope [15]. As depicted in Fig. 3a, surface and intracellular channel proteins were detected by anti-HA immunocytochemistry before and after plasma membrane permeabilization, respectively. Quantification of surface channel protein did not reveal any difference between wild-type and mutant HCN4 (Fig. 3b). Also, the subcellular localization of HCN4 V759I showing an accumulation of protein in a juxtanuclear compartment previously identified as the endocytic recycling compartment [15] did not differ from wild-type channels. Thus, both channel processing and cell surface trafficking of HCN4 was not impaired by V759I.

## Discussion

In the present study, a recently described genetic variant of the cardiac pacemaker channel HCN4 has been identified in a patient diagnosed with a putatively familial form of SSS. Recombinant investigation of this variant, which translates into HCN4 V759I, did not reveal any obvious defects, neither in HCN4 channel gating nor in its trafficking to the cell surface. Our observation allays a previous classification of the HCN4 V759I variant as being putatively pathogenic, which was based on genetic association studies; it therefore strongly emphasizes an imperative of functional studies for serious interpretation of genetic findings [1, 30].

The herein reported patient presented as a typical case of idiopathic SSS without any evidence for congenital or acquired structural heart disease, metabolic disorder, or drug-induced SSS. Because of the positive family history of SAN dysfunction, we set out to sequence the patient’s HCN4 gene, coding for the primary cardiac pacemaker channel subunit in human SAN [4]. The genetic analysis yielded the non-synonymous missense substitution of c.2275G>A, which is reported on the Exome Variant Server at a frequency of 0.6% in a European population and at 0.1% in an African American population making it a rare allele [12, 29]. The amino acid V759 is highly conserved in HCN to the level of reptile showing a high Genomic Evolutionary Rate Profiling (GERP) score of 3.45. Furthermore, the affected residue is located in the distal cytoplasmic C-terminus of HCN4, which is a known trafficking determinant of HCN channels [15] and in direct neighborhood of the cyclic nucleotide–binding domain (CNBD), which is an important gating determinant of HCN channels [33]. Therefore, we sought to characterize both gating and trafficking of the HCN4 V759I variant in more detail.

Recombinant expression of mutant channels in *Xenopus* oocytes did not reveal any obvious abnormalities in their gating behavior. Particularly, voltage-dependence of activation, its modulation by cAMP, and its kinetics, three major determinants for channel availability in SAN impulse generation, remained unchanged in mutant compared to wild-type HCN4 channels. They were in good agreement with the biophysical properties of HCN4 reported for the same heterologous expression system [7]. In isolated cases of HCN channelopathies, it has been shown that only heteromeric assembly of mutant and wild-type channels disguises gating defects, which do not show up in the homomeric assembly of mutant channels [11]. In order to mimic the patient’s heterozygous genotype for the identified variant, we hence co-expressed mutant and wild-type HCN4 channels. However, again no gating phenotype could be detected in our electrophysiological recordings. Stimulated by the fact that HCN4 V759I has been associated with malignant epilepsy [29] and that HCN4 channels are also expressed in the brain [3]; we finally investigated the functional interaction of mutant channels with the brain auxiliary subunit of HCN channels, PEX5R/Trip8b [37]. PEX5R binds to different C-terminal regions of HCN channels including their CNBD and antagonizes the effects of cAMP on channel gating [6]. In both mutant and wild-type HCN4 channels co-expressed with PEX5R, respectively, we observed a negative shift in voltage-dependence and an increase in time constants of activation, both to a similar extent and in good agreement with the published literature for wild-type channels [37]. Thus, also auxiliary PEX5R gating of neuronal HCN4 channels is not affected by the V759I mutation.

Not only the gating properties but also the number of ion channels on the cell surface eventually determine the transmembrane current at a given driving potential. In line, many channelopathies have been identified, in which the processing and trafficking of ion channel proteins are impaired [5]. As V759I is located in the cytoplasmic C-terminal domain distal of the CNBD, which is important for HCN channel trafficking [15], a final set of experiments investigated cell surface expression of HCN4 variant channels. In order to exclude possible differences in protein processing in heterologous expression systems, we performed these experiments in the rather native expression system of dissociated cardiomyocytes transduced by viral gene transfer. The protein amount of HCN4 V759I channels arriving on the cell surface and quantified by extracellular epitope tagging [15] did not differ from wild-type channels, indicating no difference in processing and trafficking of the mutant channel protein.

Given the available electrophysiological and cell biological readouts, we did not observe any obvious phenotype of HCN4 V759I. Despite good agreement with the mammalian secretory pathway of protein transport, despite of providing a plasma membrane phospholipid composition and canonical signal transduction pathways comparable to the mammalian system, which has enabled seminal work on ion channel processing and gating [17, 20, 23, 24, 36, 37], the heterologous expression system of *X. laevis* oocytes may concededly at best approximate native conditions. Albeit HCN channel family members are known to heteromerize, we did not pursue analyzing the assembly of HCN4 V759I with other HCN isoforms, as the determinants of channel tetramerization were previously localized to the N-terminus, C-linker, and CNBD domains, upstream of residue V759 [21, 28, 35]. We did neither exclude that a mutation in another ion channel gene known to be less frequently involved in SSS pathogenesis, i.e., SCN5A or KCNE2 [18, 23], exists in the here described family nor that the identified variant affects interaction of HCN4 with putative auxiliary subunits, which might worsen clinical symptoms. However, as most cases of SSS seem to be far more complex than being deducible from a single channelopathy [17, 20], also in the here presented case a failure of multiple factors including ion fluxes, gap junction properties, and signal transduction pathways, which all contribute to SAN automaticity, might be causative.

In summary, we conclude that the here described genetic variant of HCN4 resulting in a missense mutation V759I of rather small physicochemical difference (Grantham score 29) is not sufficient to cause the SSS diagnosed in the index patient. Our finding that the patient’s mother also suffering from SAN dysfunction with the need for pacemaker implantation does not carry the variant HCN4 allele most convincingly supports this view. The study once more underscores the necessity of functional studies for a careful and sophisticated interpretation of genetic association, not only in the context of hereditary cardiac arrhythmias [10].

## Data Availability

The datasets used and/or analyzed during the current study are available from the corresponding author on reasonable request.
